# Evaluation of Dosing Time-Related Anti-Hypertensive Efficacy of Valsartan in Patients with Type 2 Diabetes

**DOI:** 10.3109/10641963.2010.503306

**Published:** 2010-12-19

**Authors:** Katsunori Suzuki, Yoshifusa Aizawa

**Affiliations:** ^a^ Division of Endocrinology and Metabolism, Saiseikai Niigata Daini Hospital, Niigata, Japan; ^b^ Division of Cardiology, Niigata University Graduate School of Medical and Dental Sciences, Niigata, Japan

**Keywords:** valsartan, anti-hypertensive therapy, dose timing, morning hypertension, urinary albumin excretion

## Abstract

The aim of this study was to evaluate which administration timing of valsartan provides satisfactory blood pressure (BP) control, once daily in the morning, once daily in the evening, or twice daily in total 160 mg. Hypertensive patients with mild-to-moderate diabetic nephropathy were enrolled, but those with more than three anti-hypertensive agents, renal insufficiency (serum creatinine ≥ 3 mg/ dL), or hepatic insufficiency were excluded. They were randomized to receive valsartan 160mg once daily in the morning, valsartan 160 mg once daily in the evening, or valsartan 80 mg twice daily for 12 weeks according to a three-period crossover design. Office blood pressure (OBP), home blood pressure (HBP) self-measured by patients, and urinary albumin excretion adjusted by creatinine excretion (UAE) were measured every 12 weeks. In 34 patients, (male: 18, mean age: 57.5 ± 10.3), valsartan with ether all administration timing demonstrated significant reductions in OBP and HBP compared to baseline: valsartan 160 mg once daily in the morning: −12.2/−9.5 mmHg (p < 0.01); valsartan 160 mg once daily in the evening: −14.2/−10.3 mmHg (p < 0.01); valsartan 80 mg twice daily: −15.0/−10.2 mmHg (p < 0.01) There was no statistically significant differences in a decrease in OBP and HBP, and reduction of UAE among three administration timing. In conclusion, these data indicate that the efficacy on BP-lowering does not depend on administration timing of valsartan in patients with diabetic nephropathy.

## INTRODUCTION

It has been increasingly recognized that diabetic patients with hypertension are at a very high risk of cardiovascular disease. It is, thus, reported that diabetic patients have a 2- to 4-fold higher risk of cardiovascular disease than nondiabetic patients, with a further 2- to 3-fold increased risk of cardiovascular disease in the presence of hypertension (1, 2). In addition, concomitant hypertension results in progression of diabetic nephropathy (3).

In the Guidelines for the Management of Hypertension in 2009, by the Japanese Society of Hypertension (JSH2009) (4), diabetic patients are classified as a high-risk group, with a target blood pressure (BP) of <130/80 mmHg. For a selection of antihypertensive drugs for hypertensive patients with diabetes, renin-angiotensin (RA) system inhibitors (angiotensin-converting enzyme [ACE] inhibitor and angiotensin receptor blocker [ARB]) are recommended as first-line therapy in consideration of effects on glucose/lipid metabolism as well as prevention of complications.

Given a reference value for home BP of 135/85 mmHg, morning hypertension is defined in the JSH2009 as morning BP (mean) of ≥135/85 mmHg, despite the absence of a consensus definition for morning hypertension (4). The diurnal variation of BP is also characterized by an increase from nighttime to early morning as physiological adaptation (5). Cardiovascular events occur more frequently in the early morning, and morning BP is significantly correlated with the overall cardiovascular risk involving the brain, heart, and kidney, indicating that it is important to control morning hypertension (6).

In the JSH2009, it is recommended to suppress more morning hypertension based on 24-h control of BP, including nocturnal BP, and thereby inhibit cardiovascular events more effectively. For this purpose, it is essential to use long-acting antihypertensive drugs that remain effective for 24 h. When morning BP is still high, it is recommended to divide the dose between morning and evening or to take the drug at bedtime or after dinner.

In spite of this recommendation, few studies have been conducted to evaluate the effect of dosing time or frequency of antihypertensive drugs on morning BP. In this study, we evaluated the dosing time- or dosing frequency-related difference in the efficacy of valsartan, an ARB, at the maximum daily dose of 160 mg in hypertensive patients with type 2 diabetes.

## METHODS

Hypertensive patients with type 2 diabetes under treatment in our hospital, who met the following criteria, were included in the study: stable HbA1c during at least 3 months of antidiabetic treatment; and clinic BP of ≥130/80 mmHg as the target BP recommended in the JSH2009. These patients were randomized to receive valsartan at a dose of 160 mg once after breakfast (once in the morning), or once after dinner (once in the evening), or at a dose of 80 mg once after breakfast and once after dinner (twice daily in the morning and evening), in a crossover manner.

**Figure 1.**
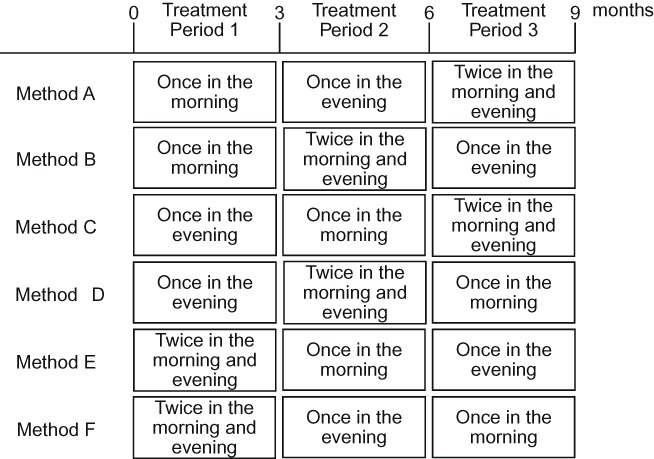
Combinations of regimens of valsartan.

Based on these three regimens, the following six combinations of regimens were administered (6 × 6 Latin square: Figure 1): Method A: once in the morning→once in the evening→twice daily in the morning and evening; Method B: once in the morning→twice daily in the morning and evening→once in the evening; Method C: once in the evening→once in the morning→twice daily in the morning and evening; Method D: once in the evening→twice daily in the morning and evening→once in the morning; Method E: twice daily in the morning and evening→once in the morning→once in the evening; and Method F: twice daily in the morning and evening→once in the morning→once in the evening. The duration of treatment with valsartan was 3 months per period (Treatment period I, Treatment period II, and Treatment period III), with no washout between the treatment periods. Six patients were allocated to each combination of regimens.

Patients were asked to measure home morning BP starting from before commencement of the study, and to submit a 7-day home BP log during the observation period and at the final visit of each treatment period (3 months after the start of each treatment period). BP was to be measured only once and recorded in a diary after 1 to 2 min of rest in a sitting position following urination and before breakfast prior to administration of valsartan. However, home evening BP was not regularly measured. Home BP was measured using an HEM-762 Fuzzy (Omron Corp., Tokyo, Japan) digital automatic sphygmomanometer, and clinic BP was measured with the fourth Korotkoff sound as a reference pressure using a mercury manometer. Patients were also asked to record a daily administration of valsartan in order to confirm the drug compliance.

The change in BP after each regimen of valsartan was assessed based on clinic BP as well as home BP (morning BP). During the observation period and each treatment period, body weight and HbA1c, as well as albumin in urine, were measured. The first morning urine was used as a sample for urinary albumin excretion (UAE), which was adjusted by creatinine concentration in urine.

The following patients were excluded from the study: those who received at least three antihypertensive drugs; those with renal impairment (serum creatinine: ≥3 mg/dL); those with serious hepatic dysfunction; diabetic patients with uncontrolled blood glucose; and those with acute cardiovascular events within 3 months. Patients with abdominal artery bruit were also excluded as doubtable renovascular hypertension. This study protocol was approved by the Institutional Review Board in our hospital and informed consent was obtained from all participating patients.

Values are expressed as mean ± standard error. Statistical analysis was performed by analysis of variance (ANOVA) and Bonferoni test as post-hoc analysis, with a two-tailed significance level of 5%. Urinary albumin excretion was skewed to higher values and was presented with medians and interquartile ranges (25th and 75th percentiles). As for statistical analysis of UAE, the values were transformed to natural logarithms.

## RESULTS

Of 36 patients enrolled, two patients failed to attend at the midpoint of the study, and the remaining 34 patients completed all regimens. Of the 34 patients, 5, 6, 6, 6, 6, and 5 patients were allocated to Methods A, B, C, D, E, and F, respectively, combinations of regimens of valsartan as described in Figure 1. Patient background data are listed in Table 1. The average age was 57.5 ± 1.8 years, and 12 patients had complication of diabetes only. While four patients received concomitant antihypertensive therapy, 28 patients received monotherapy with valsartan. A few patients out of 18 male patients had an alcohol habit, several instances of alcohol consumption per month; however, no patient was an alcoholic. Also, the compliance of valsartan at each regime was well kept in accordance to a diary.

**Table 1.** Patient background data

Age (years)	57.5 ± 1.8	
Male n (%)	18	(52.9)
BMI (kg/m^2^)	25.6 ± 0.8	
HbA1c (%)	7.19 ± 0.17	
Complication n (%)		
Diabetes	34	(100.0)
Renal disorder	12	(35.3)
Dyslipidemia	10	(29.4)
Cerebrovascular disorder	5	(14.7)
Heart disease	3	(8.8)
Antihypertensive drug n (%)		
No	28	(82.4)
Yes	4	(11.8)
Ca antagonist	6	(17.6)
ACE inhibitor	2	(5.9)
α-blocker	1	(2.9)
Antidiabetic therpy n (%)		
Dietary therapy only	4	(11.8)
Drug therapy	28	(82.4)
Biguanide	12	(35.3)
Sulfonylurea	13	(38.2)
Thiazolidine	2	(5.9)
α-GI	4	(11.8)
Insulin	18	(52.9)
Clinic blood pressure (mmHg)		
Systolic	147.0 ± 2.1	
Diastolic	89.6 ± 1.6	
Home morning blood pressure (mmHg)		
Systolic	147.0 ± 2.6	
Diastolic	84.8 ± 1.6	

As shown in Table 2, body weight and HbA1c were stable with no significant change across the treatment periods, and there was no significant difference in clinic or home BP across the treatment periods. Since there seemed to be no cumulative change dependent on the duration of treatment with valsartan, the change in BP was compared among the regimens of valsartan.

**Table 2.** Changes in variables across the treatment periods

	Treatment period I	Treatment period II	Treatment period III	p-Value (ANOVA)
Body weight (kg)	67.3 ± 2.4	67.3 ± 2.3	67.4 ± 2.3	0.9397
HbA1c (%)	7.38 ± 0.22	7.35 ± 0.19	7.19 ± 0.22	0.2445
Clinic blood pressure (mmHg)				
Systolic	134.6 ± 2.6	132.0 ± 2.0	132.5 ± 2.3	0.4802
Diastolic	79.2 ± 2.0	79.5 ± 1.7	79.8 ± 2.0	0.9548
Home morning blood pressure (mmHg)				
Systolic	136.3 ± 3.1	133.9 ± 2.7	137.9 ± 2.9	0.2840
Diastolic	78.8 ± 1.9	78.6 ± 1.9	80.9 ± 2.0	0.2105

**Figure 2.**
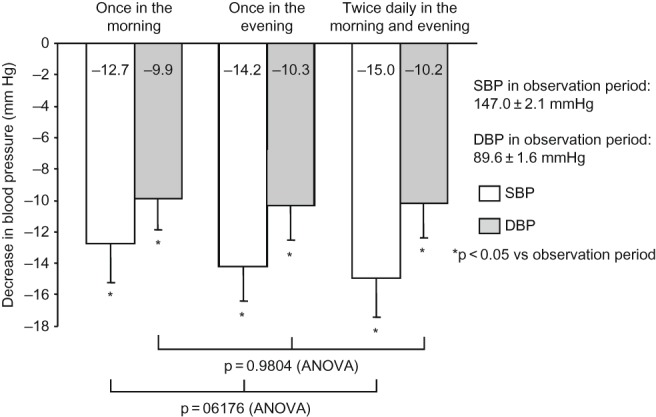
Decrease in clinic BP. During all regimens of valsartan, BP was significantly decreased from the observation period, but with no significant difference among the regimens. Comparison was performed by one-way ANOVA. Abbreviations: SBP - systolic blood pressure; DBP - diastolic blood pressure.

**Figure 3.**
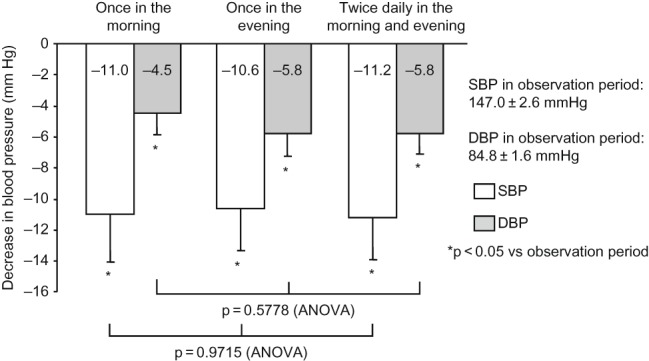
Decrease in home morning BP. During all regimens of valsartan, BP was significantly decreased from the observation period, but with no significant difference among the regimens. Comparison was performed by one-way ANOVA. Abbreviations: SBP - systolic blood pressure; DBP - diastolic blood pressure.

During all regimens of valsartan, BP was significantly decreased from the observation period, with a clinic BP of 147.0 ± 2.1/89.6 ± 1.6 mmHg and a home morning BP of 147.0 ± 2.6/84.8 ± 1.6 mmHg (Table 3). As shown in Figures 2 and 3, the decrease in BP from the observation period was not significantly different among the regimens of valsartan in terms of clinic or home morning BP.

**Table 3.** Change in BP among the regimens of valsartan

	Observation period	Once in the morning	Once in the evening	Twice daily in the morning and evening	p-Value (ANOVA)
Clinic blood pressure (mmHg)					
Systolic	147.0 ± 2.1	134.3 ± 2.2	132.8 ± 2.0	132.0 ± 2.6	<0.0001
Diastolic	89.6 ± 1.6	79.7 ± 2.1	79.3 ± 1.9	79.4 ± 1.8	<0.0001
Mean	108.7 ± 1.4	97.9 ± 1.9	97.2 ± 1.5	97.0 ± 1.6	<0.0001
Home morning blood pressure (mmHg)					
Systolic	147.0 ± 2.6	136.0 ± 3.0	136.4 ± 3.0	135.7 ± 2.7	<0.0001
Diastolic	84.8 ± 1.6	80.3 ± 1.9	79.0 ± 2.0	79.0 ± 1.8	0.0002
Mean	105.5 ± 1.6	98.9 ± 2.0	98.1 ± 2.1	97.9 ± 1.9	<0.0001

*p 0.05 vs. observation period.

Of 30 patients with a clinic BP of ≥140/90 mmHg in the observation period, 16 patients (53.3%) who received valsartan once in the morning, 20 patients (66.7%) who received valsartan once in the evening, and 18 patients (60.0%) who received valsartan twice daily in the morning and evening achieved a BP of <140/90 mmHg after treatment. Similarly, seven patients (23.3%), eight patients (26.7%), and nine patients (30.0%) achieved a BP of <130/80 mmHg, respectively. The proportion of patients who achieved either target BP was not significantly different among the regimens of valsartan (p = 0.5726 and p = 0.8430, respectively).

Of all 34 patients with a home BP of ≥125/75 mmHg in the observation period, eight patients (23.5%) who received valsartan once in the morning, seven patients (20.6%) who received valsartan once in the evening, and five patients (14.7%) who received valsartan twice daily in the morning and evening achieved a BP of <125/75 mmHg after treatment, showing no difference among the regimens of valsartan (p = 0.6398).

**Figure 4.**
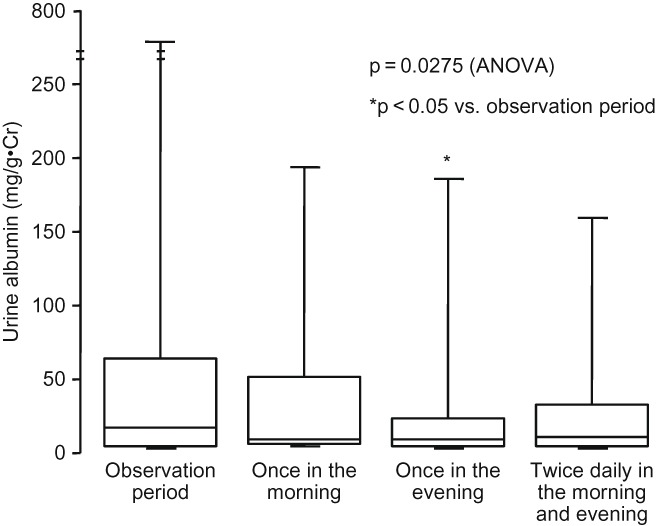
Change in urine albumin. During all regimens of valsartan, urine albumin was decreased from the observation period, with a significant difference after administration once in the evening compared to the value in the observation period. Comparison was performed by one-way ANOVA and Bonferoni test as post-hoc analysis.

Urinary albumin excretion was decreased after treatment with valsartan, showing no difference among the regimens of valsartan despite a significant decrease after administration once in the evening compared to the values in the observation period (Figure 4). In 12 patients with UAE ≥30 mg/g·Cr in the observation period, UAE tended to be decreased from 124.2 (801.0, 53.5; 25th and 75th percentiles) mg/g·Cr in the observation period to 997.7 (320.2, 27.2), 75 .0(276.8, 20.8), and 74.1 (248.0, 26.8) mg/g·Cr after administration once in the morning, once in the evening, and twice daily in the morning and evening, respectively (p = 0.0275: ANOVA).

No valsartan-related adverse event, orthostatic hypotension, dizziness, or new onset of ischemic heart disease, was found even in the period with valsartan 160 mg once in the day.

## DISCUSSION

In hypertensive patients with type 2 diabetes who could not achieve the target BP (<130/80 mmHg in clinic BP), the antihypertensive efficacy of valsartan at a daily dose of 160 mg was compared among three regimens (160 mg once after breakfast, 160 mg once after dinner, or 80 mg twice after breakfast and dinner). During all regimens, clinic and home BP was significantly decreased, with no difference in the decrease among the regimens. In addition, UAE in the first morning urine, which was measured at the same time, was decreased during all regimens, but significantly only after administration once in the evening compared with UAE at the observation period.

In healthy people, the diurnal variation in BP is characterized by an increase from awakening in the morning to the maximum level before noon, followed by a decrease during physical inactivity to the minimum level during nocturnal sleep (5). The post-awakening increase in BP may be due to sympathetic activation, and preawakening pressor response in the early morning is called morning surge. Cardiovascular events, such as stroke and myocardial infarction, occur more frequently in the morning, indicating that cardiovascular events are related to morning surge (7). In the JSH2009, it is stipulated that more appropriate drugs should be selected for antihypertensive therapy based not only on clinic BP, but also diurnal variation in BP.

The RA system, a production pathway of physiologically active substance angiotensin II, is a major factor regulating BP. In hypertensive patients, as in normotensive individuals, the diurnal variation in BP is characterized by a peak level in the early morning associated with a preawakening increase in renin secretion due to sympathetic activation, and a minimum level at midnight Angiotensin II follows a similar time course (8, 9), suggesting that not only sympathetic activation but also angiotensin II are profoundly involved in pressor response at awakening.

Among the antihypertensive drugs, ARBs, which inhibit the RA system, are widely used in clinical practice for their potent antihypertensive effect and organ-protective effect as well as good tolerability (10–12). Given the above-mentioned diurnal variation in BP and renin secretion, the antihypertensive efficacy of ARBs may be affected by dosing time. In particular, the persistence of an antihypertensive effect may be the most susceptible. In many hypertensive patients under antihypertensive therapy, clinic BP is well controlled, whereas the antihypertensive effect is the weakest before administration in the early morning, reflecting that morning hypertension is a blind spot in the current management of hypertension based on clinic BP (13).

Since valsartan, which was used in this study, is a once-daily drug with a half-life of 5.0 h (14), its effect on early morning BP may differ depending on whether it is administered in the morning or evening. Administration time dependent BP lowering effect of valsartan 160 mg for 3 months was previously evaluated by using 48-h ABPM (15). Valsartan reduced clinic BP independent of dosing time, in the morning or in the evening. In this study, also, no significant difference was observed in either the clinic or home morning BP among the regimens of valsartan.

Ohta compared morning BP in 10 hypertensive patients who received valsartan at a dose of 80 mg twice daily in the morning and evening or at a dose of 160 mg once daily in the morning for 1 to 2 months (16). In that study, there was no significant difference in the decrease in BP between the two regimens (from 170.5 mmHg to 134.1 mmHg and 132.6 mmHg, respectively) and no difference in the decrease in UAE or high-sensitivity C-reactive protein, although a dosing schedule was not a crossover manner, i.e., twice-daily dosing followed by once-daily dosing. While Ohta did not evaluate the decrease in BP after once-daily dosing in the evening, Hirose and Nakajima studied once-daily dosing in the morning and once-daily dosing in the evening (17). The change in home BP was compared after administration of valsartan at a dose of 80 mg (five patients) or 160 mg (26 patients) once daily in the morning or evening, showing that while bedtime BP was not different between the two regimens, morning BP was decreased more markedly after administration in the evening than after administration in the morning (from 150.9 mmHg to 136.6 mmHg and 144.5 mmHg, respectively). Yamagishi compared home BP after administration of valsartan at a dose of 80 mg once in the morning or evening, reporting that both bedtime and morning BP was decreased more efficiently after administration in the evening (18). The last two studies showed that the decrease in BP after administration of valsartan at a dose of 80 or 160 mg once daily was affected by dosing time, and was greater after administration in the evening in terms of morning BP only.

These findings were not supported by the present study, in which home morning BP was not different after administration once daily in the morning, once daily in the evening, or twice daily in the morning and evening. This may be explained in part by the fact that the effect of order was not eliminated in those noncrossover studies, except for the study by Hirose and Nakajima (17). In all of those studies, moreover, the duration of each regimen of valsartan was as short as less than 8 weeks (or 2 months), different from that (3 months) in the present study. Kasayuki reported that while the antihypertensive efficacy was maintained after 3 months or more of treatment with valsartan in patients who achieved a decrease of ≥20 mmHg after 1 month of treatment (responder group), BP was further decreased to the level in the responder group after 3 months of treatment in patients who could not achieve a decrease of ≥20 mmHg after 1 month of treatment (19). These results indicate that the antihypertensive effect of valsartan, which usually shows a good antihypertensive effect after 2 or 4 weeks of treatment, is sometimes progressive and requires 3 months to reach a stable level, highlighting the need for long-term observation with an adequate duration of treatment even in crossover studies, as well as the significance of the present 9-month study with 3-month regimens.

From another view, the absorption of valsartan is affected by food intake (20). This means that the BP-reducing effect of valsartan may be varied by seasonal food. However, it was reported that long-term administration of valsartan provided stable antihypertensive efficacy for 52 weeks (21). Although patients administrated valsartan after meals in this study, the variation of valsartan absorption may be neglected.

In the present study, the lack of differences among the regimens may also be attributed to concomitant diabetes. In diabetes, formation of prorenin, a precursor of renin, in the renal collecting tubule is increased (22). Prorenin is bound to the (pro)renin receptor to activate the formation of angiotensin I from angiotensinogen, with approximately 4-fold greater angiotensin I-converting activity than renin itself (23), indicating that the resultant excessive formation of angiotensin II may be involved in the development of nephropathy and/or retinopathy in diabetes. Although the plasma renin activity and angiotensin II concentration were not measured in the present study, the physiological effect of angiotensin II formed due to a diabetes-induced increase in prorenin secretion may have been inhibited during the day after administration in the morning, resulting in no difference in the antihypertensive efficacy.

In diabetes or nephropathy, a dipper BP profile with less of a decrease in nocturnal BP is often observed (24). Dipper hypertension is often associated with proteinuria (albuminuria) due to increased glomerular filtration, suggesting correlation with increased prorenin secretion in diabetes. It is considered that nocturnal BP was decreased more markedly after administration once daily in the evening, and the percentage decrease in urine albumin was correlated with nocturnal systolic blood pressure (SBP) after administration in the morning or evening (25). In the present study, valsartan caused no significant change in body weight or HbA1c, but decreased urine albumin. While there was no difference in the decrease in BP among the regimens of valsartan, but urine albumin excretion was significantly decreased after administration in the evening, suggesting that inhibition of the nocturnal activation of an RA system in the kidney may be significant.

Given the causes of morning hypertension and albuminuria, RA system inhibitors may be therapeutic options for hypertensive patients with type 2 diabetes. It was shown that antihypertensive therapy with valsartan, an RA system inhibitor, was effective in decreasing BP and UAE after once-daily dosing, irrespective of dosing time. Since the inhibitory effect of antihypertensive therapy, which is administered on a long-term basis, on cardiovascular events was shown in the Jikei Heart Study and the Kyoto Heart Study (26, 27), the inhibitory effect of valsartan on morning hypertension may be one of the mechanisms of inhibition of cardiovascular events. It is therefore concluded that while valsartan significantly decreases BP at a daily dose of 160 mg irrespective of dosing time (once-daily dosing in the morning or evening) or dosing frequency (twice daily in the morning and evening), once-daily dosing in the evening should be selected for patients with albuminuria.

**Declaration of interest** The authors report no conflicts of interest. The authors alone are responsible for the content and writing of the paper.
